# Supplementary material for: Avoiding “conflicts of interest”: a computational approach to scheduling parallel conference tracks and its human evaluation
Source: PeerJ Comput Sci. 2019 Nov 11;5:e234. doi: 10.7717/peerj-cs.234 (PMC7924486; doi:10.7717/peerj-cs.234)
Supplement: Appendix S1 [file peerj-cs-05-234-s001.pdf]

Manual Schedule

1 Saturday, May 23, 8:30 - 9:45

Session 1

8.30 - 8:45 Skull integration and modularity in five toad species of the Rhinella granulosa group

8:45 - 9:00 Exceptional avian herbivores: Multiple origins of herbivory in the bird order Anseriformes and its correlation with beak shape and body mass

9:00 - 9:15 Lizard scales in an adaptive radiation: non-random variation of scale size follows climatic and structural habitat diversity in Anolis lizards

9:15 - 9:30 Quantification of Coiling Patterns in Gastropod Shells and Evaluation of Functional Traits

9:30 - 9:45 Taking many-to-one to the next level: decoupled evolution in an ultrafast prey capture mechanism

Session 2

8.30 - 8:45 Developmental Mechanisms for Novel Morphological Evolution: Origin and Diversification of the Avian Skull

8:45 - 9:00 Late evolutionary origin of modern bird flight inferred from shoulder allometry

9:00 - 9:15 Neotenous feather replacement facilitates loss of flight in birds

9:15 - 9:30 A new island rule for birds: evolution towards flightlessness

9:30 - 9:45 PHYLOGENY AND FORELIMB DISPARITY IN WATERBIRDS

Session 3

8.30 - 8:45 C. elegans harbors pervasive cryptic genetic variation for embryogenesis

8:45 - 9:00	Reef-specific patterns of osmotic response in larval and adult eastern oysters, Crassostrea virginica, from a single estuary
9:00 - 9:15	Breaking the mold: the effects of mutations on phenotypic covariation in the fruit fly wing
9:15 - 9:30	Genetic architecture of rapid and extreme body size evolution in an island population of house mice
9:30 - 9:45	Environmental effects on genetic covariances
	**Session 4**
8.30 - 8:45	Conserved Core Genes are under Positive Selection in a Long-Term Escherichia coli Evolution Experiment
8:45 - 9:00	Intragenic epistasis on adaptive dynamics at the gene couch potato
9:00 - 9:15	Comparative genomics sheds light on the evolution and function of the Highly Iterative Palindrome -1 motif in Cyanobacteria
9:15 - 9:30	Functional analysis of the B gene homolog PISTILLATA reveals novel regulatory interactions controlling stamen identity in Aquilegia coerulea
9:30 - 9:45	Invade, co-opt, and swap: Evolution of G1/S cell cycle control in Fungi and other eukaryotes
	**Session 5**
8.30 - 8:45	Increased egg viability, male mating ability and mating frequency evolve in populations of D. melanogaster selected for resistance to cold shok
8:45 - 9:00	The evolution of fur colour: a marsupial perspective
9:00 - 9:15	Survival in a cutthroat world: experimental estimation of natural selection on stickleback armor.
9:15 - 9:30	Is it time to abandon the holey fitness landscape metaphor?
9:30 - 9:45	When field experiments yield unexpected results: Lessons learned from measuring selection in White Sands lizards
	**Session 6**
8.30 - 8:45	Can intralocus sexual conflict explain the maintenance of alternative reproductive tactics?

8:45 - 9:00	The multifaceted role of mating system on genome evolution.
9:00 - 9:15	Differential gene expression in ovarian tissue of sexual vs. asexual freshwater snails
9:15 - 9:30	Congruent phenotypic and transcriptomic responses to testosterone in both sexes:implications for the evolution of endocrine-mediated sexual dimorphism
9:30 - 9:45	Genetics of polymorphic male-male copulatory behavior in C. elegans
	**Session 7**
8.30 - 8:45	How to train your symbionts: the dynamics of domestication
8:45 - 9:00	Experimental evolution of reduced antagonism: a role for host-parasite
9:00 - 9:15	Examining the presence of a geographic mosaic of coevolution in the walnut aphid biological control system
9:15 - 9:30	Evolving virulence and defense in a symbiotic community.
9:30 - 9:45	How Nonadditivity of Fitness Impacts Alters Selection for Resistance in a Multiple-Herbivore Community
	**Session 8**
8.30 - 8:45	Genomic response to 30-years of selection for increased lifespan reveals increased immunity as correlated trait
8:45 - 9:00	EVOLUTION OF INCREASED ADULT LONGEVITY IN DROSOPHILA MELANOGASTER POPULATIONS AS CORRELATED RESPONSE FOR ADAPTATION TO LARVAL CROWDING
9:00 - 9:15	Life-history, Selection and Effective Population Size shaping Evolution during Colonization " Lessons from Drosophila melanogaster.
9:15 - 9:30	Genetic basis of ageing evolution under differential extrinsic mortality in a nematode
	**Session 9**
8.30 - 8:45	Interactions between host phylogeny and biogeography structure sponge-associated microbial communities

8:45 - 9:00	Host evolution and ecology govern community assembly of the gut microbiome in lemurs
9:00 - 9:15	Holarctic biogeography of a widespread host-symbiont association
9:15 - 9:30	Exploring patterns of symbiont diversity in natural pea aphid populations
9:30 - 9:45	Environmental context matters: the impact of microbial symbiont on invasive insect host Megacopta cribraria is mediated by host plant
	**Session 10**
8.30 - 8:45	Auto-toxicity the evolution of the muscular voltage-gated sodium channel in Phyllobates poison frogs
8:45 - 9:00	A native root herbivore drives the evolution of defensive latex metabolites in nature
9:00 - 9:15	Transcriptome phylogeny and evolution of host chemical sequestration within the lichen moths (Insecta: Lepidoptera: Erebidae)
9:15 - 9:30	Genetic basis of alkaloid resistance in harlequin toads and poison frogs
9:30 - 9:45	Sea slugs have their cake and eat it too: a phylogenetic analysis of sponge-eating nudibranchs and the defense chemicals they take and reuse
	**Session 11**
8.30 - 8:45	Is self-pollination an evolutionary dead end? The evolution of mating systems in Erythranthe section Paradantha (Phrymaceae)
8:45 - 9:00	How does pollination mutualism affect the evolution of prior self-fertilization? A model
9:00 - 9:15	The breakdown of self-incompatibility in a range expansion
9:15 - 9:30	Does separation between sexual organs affect mating? A case study from the alpine primrose Primula halleri
	**Session 12**
8.30 - 8:45	Why is Madagascar special? Diversification patterns in pelican spiders (Archaeidae)

8:45 - 9:00	Diversification and Speciation in the Ethiopian Highlands: Insights from a Radiation of Endemic Frogs
9:00 - 9:15	The total inventory of Cuatro CiÃ©negas (Coahuila, Mexico): Patterns and evolutionary causes of high diversity of an oligotrophic aquatic ecosystem
9:15 - 9:30	The tangled evolutionary histories of Madagascar's small mammals
9:30 - 9:45	Zoogeography of genus Salvelinus in Kamchatka Peninsula
	**Session 13**
8.30 - 8:45	Recent divergence in fungal populations
8:45 - 9:00	Multi-trait divergence driven by predation environment causes immigrant inviability in Brachyrhaphis fishes
9:00 - 9:15	Mechanisms for the evolution of seasonal timing in incipient species of Ostrinia moths
9:15 - 9:30	Genomic divergence of putatively adaptive genes along an altitudinal gradient in the common yellow monkeyflower, Mimulus guttatus.
9:30 - 9:45	An intraspecific gradient from C3 to C4 photosynthesis
	**Session 14**
8.30 - 8:45	Invasion and hybridization of the highly aggressive introduced reed, Phragmites australis, in the York River watershed
8:45 - 9:00	Life History and Behavior in a Primate Hybrid Zone
9:00 - 9:15	What is the link between transmission ratio distortion and sterility in Mimulus hybrids?
9:15 - 9:30	Paternal learning of a phenotype-matching trait promotes speciation at secondary contact, but not the spread of a new local adaptation

Automated Schedule

1 Saturday, May 23, 8:30 - 9:45

	**Session 1**
8.30 - 8:45	Evolution of a mating preference for a trait used in intrasexual competition in genetically monogamous populations
8:45 - 9:00	Sexually selected traits and genotype by environment interactions
9:00 - 9:15	The effect of sampling bias on the heritability of preference and the strength of sexual selection
9:15 - 9:30	The contribution of genes and the environment on traits important for pre and post copulatory reproductive success in the cactus bug Narnia femorata
9:30 - 9:45	A novel application of proteomics to quantify adaptive responses to sperm competition
	**Session 2**
8.30 - 8:45	Evolutionary genetics of the selfish Segregation Distorter complex
8:45 - 9:00	Phylogeographic model selection using approximated likelihoods
9:00 - 9:15	Experimental Evolution of Increased Size and Complexity of Anabaena variabilis
9:15 - 9:30	Early Evolution of the Genetic Basis for Soma in the Volvocine Green Algae
9:30 - 9:45	Using linked microsatellites to infer basic population decline parameters a case study on a Mexican relict spruce
	**Session 3**
8.30 - 8:45	Life history effects and demographic consequences of interacting QTL for flowering and seed dormancy in Arabidopsis thaliana

8:45 - 9:00	The evolution of host perception in parasitic plants of the Orobanchaceae
9:00 - 9:15	Mechanisms for the evolution of seasonal timing in incipient species of Ostrinia moths
9:15 - 9:30	Natural variation in seed germination speed of Arabidopsis thaliana complex genetic architecture and response to strong selection
9:30 - 9:45	Plasticity of seed dormancy compensates for differences in dispersal timing
	**Session 4**
8.30 - 8:45	Evolutionary history and traits not invasive status influences community assembly
8:45 - 9:00	Anchored phylogenomics and transcriptomics comparisons between two next gen data sets used for estimating deep level relationships in Lepidoptera
9:00 - 9:15	Transcriptome phylogeny and evolution of host chemical sequestration within the lichen moths Insecta Lepidoptera Erebidae
9:15 - 9:30	Sea slugs have their cake and eat it too a phylogenetic analysis of sponge eating nudibranchs and the defense chemicals they take and reuse
9:30 - 9:45	Darwin s conundrum revisited does phylogenetic distance predict invasibility
	**Session 5**
8.30 - 8:45	Population determinants of persistence migration and colonization success in a plant metapopulation
8:45 - 9:00	Genetic and phenotypic divergence in an island bird isolation by distance by colonization or by adaptation
9:00 - 9:15	Speciation and chemical differentiation in the aposematic and mimetic butterflies Melinaea
9:15 - 9:30	Computationally efficient estimation of the number of founders for colonized populations
9:30 - 9:45	Genetic and environmental contributions to a divergent plumage trait in barn swallows

Session 6

8.30 - 8:45	Cardiac myopathy and flight performance in starvation selected Drosophila or the case of the All American flies
8:45 - 9:00	Genome wide scans for signals of molecular adaptation in polar bear
9:00 - 9:15	Evolutionary genetics of pigmentation variation in natural populations of Drosophila melanogaster
9:15 - 9:30	The evolution and transcriptional connectivity of genes underlying ant division of labor
9:30 - 9:45	The genetic architecture of natural variation in abdominal pigmentation of Drosophila melanogaster females

Session 7

8.30 - 8:45	How Nonadditivity of Fitness Impacts Alters Selection for Resistance in a Multiple Herbivore Community
8:45 - 9:00	Episodic nucleotide substitutions in seasonal influenza virus H3N2 can be explained by stochastic genealogical process without positive selection
9:00 - 9:15	Evolution of elemental composition in E coli under carbon and nitrogen limitation
9:15 - 9:30	Metagenomic analysis of a ssDNA viral community
9:30 - 9:45	Endogenous hepdnaviruses bornaviruses and circoviruses in snakes

Session 8

8.30 - 8:45	A single gene affects both ecological divergence and mate choice in Drosophila
8:45 - 9:00	Identifying genes affecting both adaptive divergence and reproductive isolation in Howea palms from Lord Howe Island using RNA Seq
9:00 - 9:15	Gene family evolution and functional plasticity following whole genome duplication events in plants
9:15 - 9:30	Genomic imprints of freshwater transitions in the alewife Alosa pseudoharengus
9:30 - 9:45	Phylogenomics reveals rapid and complex evolutionary divergence and speciation in wild Solanum

Session 9

8.30 - 8:45	A genomic selection component experiment in Mimulus guttatus
8:45 - 9:00	Quantification of coiling patterns in gastropod shells and evaluation of functional traits
9:00 - 9:15	Looking for evolutionary history of nematodes on the beach to better manage their populations in the fields
9:15 - 9:30	When field experiments yield unexpected results lessons learned from measuring selection in White Sands lizards
9:30 - 9:45	Survival of the fattest Indices of body condition do not predict fitness in the brown anole Anolis sagrei

Session 10

8.30 - 8:45	Phylogenomics shows multiple human infectious lineages of Trypanosoma brucei
8:45 - 9:00	Microbiome Diversity and Dynamics under Neutral and Selective Models
9:00 - 9:15	Description of a Novel Genetic Marker for Species Identification of Freshwater Mussel Larvae Recovered from Naturally Infested Fish Hosts
9:15 - 9:30	The diversity and evolution of the primate skin microbiome how different are humans from our closest relatives
9:30 - 9:45	Distribution specificity and horizontal transmission of microbial symbionts in army ant colonies

Session 11

8.30 - 8:45	Genes vs culture song variation across an avian hybrid zone
8:45 - 9:00	A simple two locus hybrid incompatibility underlies inviability between sympatric Mimulus species
9:00 - 9:15	A phylogenetic model for measuring departures from the mutation selection balance
9:15 - 9:30	The origin of species by means of Dobzhansky Muller incompatibilities
9:30 - 9:45	Two locus hybrid incompatibilities and the introgression of adaptive alleles

	**Session 12**
8.30 - 8:45	FlatNJ A novel network based approach to visualize evolutionary and biogeographical relationships
8:45 - 9:00	Information flow through dominance network in social insect colonies
9:00 - 9:15	PASTA A new method to co estimate alignments and trees even on ultra large datasets with high accuracy and speed
9:15 - 9:30	RNAseq analysis elucidate early responses to infection in scleractinian corals
9:30 - 9:45	Reduced specialization and modularity in an intimate mutualism diversifying on young oceanic islands
	**Session 13**
8.30 - 8:45	The evolution of fur colour a marsupial perspective
8:45 - 9:00	The genetic architecture of local adaptation at fine spatial scales a case study of three montane conifer species
9:00 - 9:15	Using pooled sequencing and whole genome environmental association analyses to study local adaptation in three Alpine Brassicaceae species
9:15 - 9:30	Incongruence among classes of markers and data types in supermatrices implications for phylogenomics and Drosophila evolution
9:30 - 9:45	Local adaptation to climate within a tree species range the case of sugar pine Pinus lambertiana
	**Session 14**
8.30 - 8:45	Lizard scales in an adaptive radiation variation of scale number follows climatic and structural habitat diversity in Anolis lizards
8:45 - 9:00	Who Are the Fathers Characterizing Hybrid Origins of Parthenogenetic Aspidoscelis Lizards
9:00 - 9:15	Diversification and Speciation in the Ethiopian Highlands Insights from a Radiation of Endemic Frogs
9:15 - 9:30	Dynamic gradients of river systems mediating dispersal and vicariance of fishes
9:30 - 9:45	Convergent evolution of alternative developmental trajectories associated with diapause in African and South American killifish

Random Schedule

1 Saturday, May 23, 8:30 - 9:45

	**Session 1**
8:30 - 8:45	Dynamic gradients of river systems mediating dispersal and vicariance of fishes
8:45 - 9:00	Male-driven evolution of self-compatibility in diploid and polyploid Arabidopsis
9:00 - 9:15	Caught in the Crossfire: Genes tangled up in host-mediated transposable element defense
9:15 - 9:30	Bioenergetics of transcription-factor evolution: pleiotropic constraint, compensation and a sweet spot for hybrid incompatibility
9:30 - 9:45	Tinkering with the axial skeleton: vertebral number variation in ecologically divergent threespine stickleback populations
	**Session 2**
8:30 - 8:45	Phenotypic evolution of bat skulls, and its relationship with speciation
8:45 - 9:00	Population genomics of two ecologically disparate Fundulus hybrid zones.
9:00 - 9:15	Is genetic rescue a valid option for arid zone Acacia?
9:15 - 9:30	Ecological opportunity and ecomorphological evolution in North American canids
9:30 - 9:45	Ribozyme plasticity and molecular trade-offs can account for increasing complexity and network stability at the origin of life.
	**Session 3**
8:30 - 8:45	An ecological diversification history of marine animals

8:45 - 9:00	Patterns of gene flow and reproductive isolation in closely related species of mushroom-feeding Drosophila
9:00 - 9:15	Impact of ascertainment bias on phylogenetics of foodborne pathogen outbreaks
9:15 - 9:30	Flash signal evolution in North American Photinus fireflies
9:30 - 9:45	Towards inferring the history of life in the presence of lateral gene transfers
	**Session 4**
8:30 - 8:45	Diverging genomes of an emerging ecogenomic model using Restriction-site Associated DNA (RAD)-sequencing
8:45 - 9:00	Multi-tagged pyrosequencing reveals highly polymorphic MHC genes in the endangered San Joaquin kit fox
9:00 - 9:15	What ion channel gene duplications can tell us about the origin(s) of the nervous system
9:15 - 9:30	The contrasting roles of sexual selection during speciation with gene flow
9:30 - 9:45	Genomics of local adaptation in evolving populations of microbes
	**Session 5**
8:30 - 8:45	Target Enrichment of Ultraconserved Elements in Sky Island Frogs of the Brazilian Atlantic Rainforest
8:45 - 9:00	Strong premating reproductive isolation contributes to incipient speciation in Mimulus aurantiacus
9:00 - 9:15	Comparing Patterns of Molecular Evolution in Nuclear-Encoded Mitochondrial Genes in Sexual and Asexual Lineages of a New Zealand Freshwater Snail
9:15 - 9:30	Teams of Transcription Factors are rewired in the evolution of Ascomycota fungi
9:30 - 9:45	The evolution of semelparity and egg size
	**Session 6**
8:30 - 8:45	Phylogenomics and Next-Generation Inferences: the Future of Phylogenetics in an Era of Big Data

8:45 - 9:00	Sex-specific effects of brain size on survival under semi-natural conditions in the guppy (Poecilia reticulata)
9:00 - 9:15	Exploring patterns of symbiont diversity in natural pea aphid populations
9:15 - 9:30	Phenotypic variation and differential gene expression in Virginia opossum (Didelphis virginiana) populations inhabiting different environments.
9:30 - 9:45	Genes, mice and Vikings
	**Session 7**
8:30 - 8:45	Disentangling phylogenetic relationships complicated by polyploidy in the genus Phlox (Polemoniaceae)
8:45 - 9:00	Ancient duplication of vomeronasal receptor class 1 (V1R) genes in lemurs
9:00 - 9:15	Genomic discord at different stages of divergence with gene flow in six lineages of Holarctic ducks
9:15 - 9:30	MAC-PRF: Inferring intragenic clusters of sites under natural selection from polymorphism and divergence
9:30 - 9:45	The role of ontogeny in homology statements: case studies from morphology and phylogenomics of pancrustaceans
	**Session 8**
8:30 - 8:45	Allele changes during spore formation on the mycorrhizal fungi, Rhizophagus irregularis
8:45 - 9:00	Fine-scale analysis of a genome-wide linkage map to examine meiotic recombination in the honey bee, Apis mellifera.
9:00 - 9:15	Determining asexual versus sexual propagation in the octocoral Paramuricea using RAD sequencing
9:15 - 9:30	Temporal scaling of spontaneous mutation rates: implications for the neutral theory of molecular evolution
9:30 - 9:45	RNA-seq reveals strong evidence of positive selection and gene expression differences over 20 million years of evolution in Heteromyid rodents
	**Session 9**
8:30 - 8:45	Information flow through dominance network in social insect colonies

8:45 - 9:00	Inferring allele frequency trajectories of experimentally evolved Drosophila populations with Gaussian process models
9:00 - 9:15	Estimating phenotypic selection in an age-structured moose Alces alces population by removing transient fluctuations
9:15 - 9:30	Dissecting the physiological basis of allochronic isolation in Rhagoletis
9:30 - 9:45	Do color-assortative female preferences generate reproductive isolation in a polymorphic frog?
	**Session 10**
8:30 - 8:45	Timing the diversification of the Amazonian biota: butterfly divergences are consistent with Pleistocene refugia
8:45 - 9:00	Island and continental biogeography dynamics in the assembly of the Western Ghats (India) avifauna
9:00 - 9:15	Deterministic Evolution in Greater Antillean Boid Snakes: an Extension of the Caribbean Ecomorph Paradigm?
9:15 - 9:30	A gradual scenario for the evolution of a complex behavioral trait: migration in Tyrant Flycatchers
9:30 - 9:45	Playing smart and playing safe: the joint expression of phenotypic plasticity and diversification bet hedging
	**Session 11**
8:30 - 8:45	Effects of trait-dependent speciation and extinction on the phylogenetic placement of fossil taxa
8:45 - 9:00	Locating a selection signature inside chromosomal rearrangements for tests of adaptive divergence in Anopheles gambiae
9:00 - 9:15	Evolving virulence and defense in a symbiotic community.
9:15 - 9:30	Inferring differential introgression among Southern African White eyes
9:30 - 9:45	Centromere-associated drive and the maintenance of fitness variation in Mimulus
	**Session 12**
8:30 - 8:45	A global analysis of water and nitrogen relationship between mistletoes and their hosts: broad-scale tests of old and enduring hypotheses

8:45 - 9:00	The diversity and evolution of the primate skin microbiome: how different are humans from our closest relatives?
9:00 - 9:15	Linking neotropical biomes in a spider web
9:15 - 9:30	Recent divergence in fungal populations
9:30 - 9:45	“Fitness” has at least three incommensurable dimensions: growth, efficiency, and competitiveness
	**Session 13**
8:30 - 8:45	Evolution of a troglobitic salamander, Eurycea spelaea
8:45 - 9:00	Ecological genomics of incipient speciation in Mimulus aurantiacus
9:00 - 9:15	Empirical evidence for a continuum between semelparity and iteroparity
9:15 - 9:30	The effect of herbicide resistance on mating system in the common morning glory, Ipomoea purpurea
9:30 - 9:45	HOST BIRDS COMBAT CUCKOO MIMICRY BY EVOLVING RECOGNIZABLE EGG PATTERN SIGNATURES
	**Session 14**
8:30 - 8:45	Making sense of floral scents: floral scent in the genus Mimulus and its role in pollinator shifts
8:45 - 9:00	Phylogeography and speciation of Pitcairnia flammea (Bromeliaceae) adapted to Neotropical rock outcrops
9:00 - 9:15	Phylogeny, morphology and ontogeny of the Spikethumb Frogs (Hylidae: Plectrohyla)
9:15 - 9:30	Approaches to reducing spurious signal in phylogenomic datasets
9:30 - 9:45	Dad saves the day: biparental plastid inheritance rescues cytonuclear incompatibility

Link to the survey: https://pmanda.typeform.com/to/m3tPBi

Welcome to the survey.

Please review the conference schedule attached to the survey invitation email and select talks you would like to attend. Abstracts of the talks are in the attached abstract book.

The questions in this questionnaire will ask you to record the talks/sessions you chose to attend. The survey should take approximately 15 minutes. Thanks for participating!

start press ENTER

1→ What is your level of expertise in Evolutionary Biology? *

- A Undergraduate student
- B Graduate student/Postdoc
- C Faculty

2 → Select a talk to attend from 8:30 - 8:45 am *

Type or select an option

- Auto-toxicity the evolution of the muscular voltage-gated sodium channel in Phyllobates poison frogs
- Is self-pollination an evolutionary dead end? The evolution of mating systems in Erythranthe section Paradantha (Phrymaceae)
- Skull integration and modularity in five toad species of the Rhinella granulosa group
- Developmental Mechanisms for Novel Morphological Evolution: Origin and Diversification of the Avian Skull
- C. elegans harbors pervasive cryptic genetic variation for embryogenesis
- Conserved Core Genes are under Positive Selection in a Long-Term Escherichia coli Evolution Experiment
- Recent divergence in fungal populations
- Genomic response to 30-years of selection for increased lifespan reveals increased immunity as correlated trait
- Can intralocus sexual conflict explain the maintenance of alternative reproductive tactics?

3 → Select a talk to attend from 8:45 - 9:00 am *

Type or select an option

- Late evolutionary origin of modern bird flight inferred from shoulder allometry
- Experimental evolution of reduced antagonism: a role for host-parasite
- 4 → The evolution of fur colour: a marsupial perspective
- The multifaceted role of mating system on genome evolution.
- Diversification and Speciation in the Ethiopian Highlands: Insights from a Radiation of Endemic Frogs
- Intragenic epistasis on adaptive dynamics at the gene couch potato
- Exceptional avian herbivores: Multiple origins of herbivory in the bird order Anseriformes and its correlation with beak shape and body mass
- 5 → Life History and Behavior in a Primate Hybrid Zone
- Reef-specific patterns of osmotic response in larval and adult eastern oysters, Crassostrea virginica, from a single estuary
- Multi-trait divergence driven by predation environment causes immigrant inviability in Brachyrhaphis fishes

4 → Select a talk to attend from 9:00 - 9:15 am *

Type or select an option

- Examining the presence of a geographic mosaic of coevolution in the walnut aphid biological control system
- Holarctic biogeography of a widespread host-symbiont association
- The breakdown of self-incompatibility in a range expansion
- Neotenous feather replacement facilitates loss of flight in birds
- Mechanisms for the evolution of seasonal timing in incipient species of Ostrinia moths
- Life-history, Selection and Effective Population Size shaping Evolution during Colonization “ Lessons from Drosophila melanogaster.
- Comparative genomics sheds light on the evolution and function of the Highly Iterative Palindrome -1 motif in Cyanobacteria
- What is the link between transmission ratio distortion and sterility in Mimulus hybrids?
- Differential gene expression in ovarian tissue of sexual vs. asexual freshwater snails
- Breaking the mold: the effects of mutations on phenotypic covariation in [illegible]

5 → Select a talk to attend from 9:15 - 9:30 am *

Type or select an option

- Genetic architecture of rapid and extreme body size evolution in an island population of house mice
- Genomic divergence of putatively adaptive genes along an altitudinal gradient in the common yellow monkeyflower, Mimulus guttatus.
- Congruent phenotypic and transcriptomic responses to testosterone in both sexes:implications for the evolution of endocrine-mediated sexual dimorphism
- Is it time to abandon the holey fitness landscape metaphor?
- Evolving virulence and defense in a symbiotic community.
- Does separation between sexual organs affect mating? A case study from the alpine primrose Primula halleri
- Quantification of Coiling Patterns in Gastropod Shells and Evaluation of Functional Traits
- Functional analysis of the B gene homolog PISTILLATA reveals novel regulatory interactions controlling stamen identity in Aquilegia coerulea
- Paternal learning of a phenotype-matching trait promotes speciation at

6 → Select a talk to attend from 9:30 - 9:45 am

Type or select an option

- An intraspecific gradient from C3 to C4 photosynthesis
- Taking many-to-one to the next level: decoupled evolution in an ultrafast prey capture mechanism
- Genetics of polymorphic male-male copulatory behavior in C. elegans
- Environmental context matters: the impact of microbial symbiont on invasive insect host Megacopta cribraria is mediated by host plant
- PHYLOGENY AND FORELIMB DISPARITY IN WATERBIRDS
- Zoogeography of genus Salvelinus in Kamchatka Peninsula
- When field experiments yield unexpected results: Lessons learned from measuring selection in White Sands lizards
- Invade, co-opt, and swap: Evolution of G1/S cell cycle control in Fungi and other eukaryotes
- Sea slugs have their cake and eat it too: a phylogenetic analysis of sponge-eating nudibranchs and the defense chemicals they take and reuse
- Environmental effects on genetic covariances

7 → Select a talk to attend from 10:00 -10:15 am *

Type or select an option

- Phylogenomics shows multiple human infectious lineages of Trypanosoma brucei
- Evolution of a mating preference for a trait used in intrasexual competition in genetically monogamous populations
- The evolution of fur colour a marsupial perspective
- Cardiac myopathy and flight performance in starvation selected Drosophila or the case of the All American flies
- Population determinants of persistence migration and colonization success in a plant metapopulation
- Lizard scales in an adaptive radiation variation of scale number follows climatic and structural habitat diversity in Anolis lizards
- FlatNJ A novel network based approach to visualize evolutionary and biogeographical relationships
- Genes vs culture song variation across an avian hybrid zone
- A genomic selection component experiment in Mimulus guttatus
- Evolutionary history and traits not invasive status influences community

8 → Select a talk to attend from 10:15 - 10:30 am *

Type or select an option

- Genetic and phenotypic divergence in an island bird isolation by distance by colonization or by adaptation
- A simple two locus hybrid incompatibility underlies inviability between sympatric Mimulus species
- Identifying genes affecting both adaptive divergence and reproductive isolation in Howea palms from Lord Howe Island using RNA Seq
- Who Are the Fathers Characterizing Hybrid Origins of Parthenogenetic Aspidoscelis Lizards
- The evolution of host perception in parasitic plants of the Orobanchaceae
- The genetic architecture of local adaptation at fine spatial scales a case study of three montane conifer species
- Sexually selected traits and genotype by environment interactions
- Genome wide scans for signals of molecular adaptation in polar bear
- Phylogeographic model selection using approximated likelihoods
- Microbiome Diversity and Dynamics under Neutral and Selective Models

9 → Select a talk to attend from 10:30 - 10:45 am *

Type or select an option

- Diversification and Speciation in the Ethiopian Highlands Insights from a Radiation of Endemic Frogs
- Mechanisms for the evolution of seasonal timing in incipient species of Ostrinia moths
- Speciation and chemical differentiation in the aposematic and mimetic butterflies Melinaea
- Transcriptome phylogeny and evolution of host chemical sequestration within the lichen moths Insecta Lepidoptera Erebidae
- Experimental Evolution of Increased Size and Complexity of Anabaena variabilis
- A phylogenetic model for measuring departures from the mutation selection balance
- Gene family evolution and functional plasticity following whole genome duplication events in plants
- PASTA A new method to co estimate alignments and trees even on ultra large datasets with high accuracy and speed
- [illegible]

10 → Select a talk to attend from 10:45 - 11:00 am *

Type or select an option

- Dynamic gradients of river systems mediating dispersal and vicariance of fishes
- Incongruence among classes of markers and data types in supermatrices implications for phylogenomics and Drosophila evolution
- Metagenomic analysis of a ssDNA viral community
- When field experiments yield unexpected results lessons learned from measuring selection in White Sands lizards
- The contribution of genes and the environment on traits important for pre and post copulatory reproductive success in the cactus bug Narnia femorata
- Sea slugs have their cake and eat it too a phylogenetic analysis of sponge eating nudibranchs and the defense chemicals they take and reuse
- Genomic imprints of freshwater transitions in the alewife Alosa pseudoharengus
- Early Evolution of the Genetic Basis for Soma in the Volvocine Green Algae
- The evolution and transcriptional connectivity of genes underlying ant

11 → Select a talk to attend from 11:00 - 11:15 am *

Type or select an option

- Reduced specialization and modularity in an intimate mutualism diversifying on young oceanic islands
- Phylogenomics reveals rapid and complex evolutionary divergence and speciation in wild Solanum
- A novel application of proteomics to quantify adaptive responses to sperm competition
- Distribution specificity and horizontal transmission of microbial symbionts in army ant colonies
- Plasticity of seed dormancy compensates for differences in dispersal timing
- The genetic architecture of natural variation in abdominal pigmentation of Drosophila melanogaster females
- Survival of the fattest Indices of body condition do not predict fitness in the brown anole Anolis sagrei
- Using linked microsatellites to infer basic population decline parameters a case study on a Mexican relict spruce
- Local adaptation to climate within a tree species range the case of sugar

12 → Choose one session to attend in its entirety from 8:30 - to 9:45 am. This means that you will be attending all talks in that session. How easy was it to find a session where all the talks interested you ? *

1	2	3	4	5

Very difficult — Neutral — Very easy

13 → Choose one session to attend in its entirety from 10:00 - to 11:15 am. This means that you will be attending all talks in that session. How easy was it to find a session where all the talks interested you ? *

1	2	3	4	5

Very difficult Neutral Very easy
